# Innovative pulmonary targeting of terbutaline sulfate-laded novasomes for non-invasive tackling of asthma: statistical optimization and comparative *in vitro*/*in vivo* evaluation

**DOI:** 10.1080/10717544.2022.2092236

**Published:** 2022-07-08

**Authors:** Mohammed H. Elkomy, Shahira F. El Menshawe, Rasha M. Kharshoum, Amany M. Abdeltwab, Raghda R. S. Hussein, Doaa S. Hamad, Izzeddin Alsalahat, Heba M. Aboud

**Affiliations:** a Department of Pharmaceutics, College of Pharmacy, Jouf University, Sakaka, Saudi Arabia; b Department of Pharmaceutics and Industrial Pharmacy, Faculty of Pharmacy, Beni-Suef University, Beni-Suef, Egypt; c Department of Clinical Pharmacy, Faculty of Pharmacy, Beni-Suef University, Beni-Suef, Egypt; d Department of Clinical Pharmacy, Faculty of Pharmacy, Modern University for Technology and Information, Cairo, Egypt; e Department of Pharmaceutics, Faculty of Pharmacy, Nahda University, Beni-Suef, Egypt; f UK Dementia Research Institute Cardiff, School of Medicine, Cardiff University, Cardiff, UK

**Keywords:** Bronchial asthma, pulmonary targeting, terbutaline sulfate, novasomes, Box-Behnken design, pharmacokinetics

## Abstract

Asthma represents a globally serious non-communicable ailment with significant public health outcomes for both pediatrics and adults triggering vast morbidity and fatality in critical cases. The β_2_-adrenoceptor agonist, terbutaline sulfate (TBN), is harnessed as a bronchodilator for monitoring asthma noising symptoms. Nevertheless, the hepatic first-pass metabolism correlated with TBN oral administration mitigates its clinical performance. Likewise, the regimens of inhaled TBN dosage forms restrict its exploitation. Consequently, this work is concerned with the assimilation of TBN into a novel non-phospholipid nanovesicular paradigm termed novasomes (NVS) for direct and effective TBN pulmonary targeting. TBN-NVS were tailored based on the thin film hydration method and Box-Behnken design was applied to statistically optimize the formulation variables. Also, the aerodynamic pattern of the optimal TBN-NVS was explored *via* cascade impaction. Moreover, comparative pharmacokinetic studies were conducted using a rat model. TBN elicited encapsulation efficiency as high as 70%. The optimized TBN-NVS formulation disclosed an average nano-size of 223.89 nm, ζ potential of −31.17 mV and a sustained drug release up to 24 h. Additionally, it manifested snowballed *in vitro* lung deposition behavior in cascade impactor with a fine particle fraction of 86.44%. *In vivo* histopathological studies verified safety of intratracheally-administered TBN-NVS. The pharmacokinetic studies divulged 3.88-fold accentuation in TBN bioavailability from the optimum TBN-NVS versus the oral TBN solution. Concisely, the results proposed that NVS are an auspicious nanovector for TBN pulmonary delivery with integral curbing of the disease owing to target specificity.

## Introduction

Asthma is a common, multifactorial and almost chronic pulmonary disorder characterized by episodic or persistent symptoms of airway inflammation, bronchospasm besides hyper-responsiveness, provoking poor quality of life, uncomfortable sensation and sometimes mortality (Stern et al., 2020). The cellulosic sympathomimetic amine, β_2_-adrenergic receptor stimulant terbutaline sulfate (TBN), has been widely exploited for managing chronic bronchitis, bronchial asthma, chronic obstructive pulmonary disease (COPD) and emphysema (Gulsun et al., 2018). Systemic treatment with TBN suffers from first-pass elimination in the liver, thus it endures inadequate bioavailability of nearly 10% (Hochhaus & Möllmann, 2019). On the other hand, pulmonary drug delivery stands out as a non-invasive alternative route that directly targets the drugs into lungs (Li et al., 2018) to avoid first-pass metabolism and also to minimize undesirable systemic activity (Chaurasiya & Zhao, 2020). Actually, inhalation is a common successful approach regarding airway-related diseases such as asthma (Chaurasiya & Zhao, 2020), lung cancer (Zhang et al., 2020) and pneumonia (Yu et al., 2021). Unfortunately, conventional TBN aerosol formulations are inconvenient for patients since they display a fast pharmacokinetic profile and thus, the dose must be scheduled to be repeated every 4–6 h to maintain the bronchodilation effect (Chen et al., 2012). Accordingly, a novel efficacious strategy is desperately required to convey a sufficient dose of drug to the lung in a sustained manner. Over the years, a great attention has been grabbed to nanotechnology as a fruitful maneuver for pulmonary drug delivery (Doroudian et al., 2021). Such nano-cargos facilitate the delivery of drugs and biological materials straightway to the targeted tissues. This has evoked a promoted pharmacological impact with significantly fewer adverse effects (Hami, 2021). However, physiologic barriers in the lung such as mucus hyperproduction, mucociliary clearance, macrophages, neutrophils and subepithelial fibrosis curb the application of this therapeutic strategy (Schneider et al., 2017). Thus, the nanovector particles must comply with unique specifications comprising an adequate mass median aerodynamic diameter (MMAD) and an appropriate fine particle fraction (FPF) (Elkomy et al., 2021).

Novasomes (NVS) are a new paradigm derived from liposomes and they can be also considered a variation of niosomes. NVS are promising patented non-phospholipid vesicles with a submicron size range (100–1000 nm) (Mehanna & Mneimneh, 2020). They are composed of a mixture of cholesterol, free fatty acids and polyoxyethylene fatty acid monoesters. NVS are multi-bilayered nanovesicles with a great gage centralized core, efficiently loading more than 80% of hydrophilic or lipophilic constituents, consequently, reducing administration frequency (Atef et al., 2022). NVS have considerable exploitations in the arenas of cosmetics, personal care, nutrition, chemicals, agrochemicals, pharmaceuticals and also, they have been employed as an adjuvant for human vaccines (Abd-Elal et al., 2016). Recently, NVS have been investigated as a valuable nanoplatform for topical delivery of terconazole (Mosallam et al., 2021) and transdermal delivery of agomelatine (Tawfik et al., 2021). To our best knowledge, no systematic empirical research has been reported yet in the pulmonary delivery with NVS.

Hence, the purpose of the present study was to fabricate a combinatorial system based on TBN-NVS to be administered through the intratracheal (i.t) route for effective pulmonary delivery of TBN. Thus, a highly concentrated and stable TBN formulation with ideal physicochemical parameters and optimal nanovesicular size was determined using the Box-Behnken statistical design and the *in vitro* performances were scrutinized. In addition, the aerodynamic aspects of the tailored nanovesicles were estimated employing a cascade impactor. Furthermore, *in vivo* histopatholgical investigation was conducted to assess the irritative prospect of the optimized TBN-NVS formulation on rat lung. Finally, the pharmacokinetic profile of i.t TBN-NVS nanosuspension was compared with orally and i.t administered aqueous solution of TBN using Wistar male rats.

## Materials and methods

### Materials

Terbutaline sulfate was kindly provided by SEDICO Company (Cairo, Egypt). Cholesterol, Span 60, chloroform, methanol (HPLC grade) and dialysis bags with a molecular weight cut off of 12,000 Da were purchased from Sigma-Aldrich (St. Louis, MO, USA). Stearic acid, disodium hydrogen phosphate, potassium dihydrogen phosphate, potassium chloride and sodium chloride were purchased from El-Nasr pharmaceutical chemical company (Cairo, Egypt). All the other enrolled substances in the study were of analytical laboratory grade.

### Experimental design

To probe the impacts of the different formulation moderators on the features of TBN-NVS, Box-Behnken design was statistically employed using Design Expert® software (Version 12.0.3.0, Stat-Ease Inc. Minneapolis, MN, USA). A design matrix involving 3 causal formulation factors at 3 different levels and 15 TBN-NVS formulations were fabricated; 12 constitute the mid-points of the edges of a 3-dimensional cube, while the remainder constitute the center of the cube repeated in a triplicate. The concentrations of cholesterol (*X_1_*), Span 60 (*X_2_*) and stearic acid (*X_3_*) were the elected causal variables. The dependent response variables were entrapment efficiency percent (*Y_1_*: EE%), particle size (*Y_2_*: PS) and accumulative percentage drug release after 8 h (*Y_3_*: Q_8h_%). The levels of the independent variables (low, medium and high) were selected according to the results obtained from the preliminary experimentation as denoted in Table 1. Table 2 outlines the composition of the 15 formulations of TBN-NVS from the Box-Behnken design.

**Table 1.** Box-Behnken design for optimization of the TBN-NVS.

Factor (independent variables)	Level		
	−1	0	+1
*X_1_*: Cholesterol (mg)	20	40	60
*X_2_*: Span 60 (mg)	50	100	150
*X_3_*: Stearic acid (mg)	15	25	50
Responses (dependent variables)	Constraints		
*Y_1_*: Encapsulation Efficiency (%)	Maximize		
*Y_2_*: Particle size (nm)	Minimize		
*Y_3_*: Drug release Q_8h_ (%)	Maximize		

TBN: terbutaline sulfate; NVS: novasomes.

**Table 2.** Composition of TBN-NVS Formulations based on the Box-Behnken design and the observed response variables.

	Independent variables			Dependent variables			
Formulation	*X_1_* Cholesterol (mg)	*X_2_* Span 60 (mg)	*X_3_* Stearic acid (mg)	*Y_1_* EE (%)	*Y_2_* PS (nm)	*Y_3_* Q_8h_ (%)	PDI
F1^a^	40	100	25	68.93 ± 6.04	233.19 ± 19.46	68.18 ± 1.95	.41
F2	20	150	25	67.28 ± 2.83	311.26 ± 13.63	62.35 ± 2.79	.59
F3	40	50	15	60.31 ± 2.46	205.94 ± 6.82	78.70 ± 1.34	.51
F4	20	50	25	45.72 ± 3.23	300.83 ± 14.02	74.14 ± 0.91	.31
F5^a^	40	100	25	68.11 ± 7.21	235.10 ± 21.06	69.05 ± 2.08	.39
F6	40	150	50	69.15 ± 4.41	400.21 ± 32.86	60.47 ± 2.17	.37
F7	60	50	25	34.87 ± 3.23	314.18 ± 12.04	63.56 ± 1.19	.32
F8	20	100	15	63.07 ± 4.65	225.74 ± 17.64	76.93 ± 3.37	.59
F9^a^	40	100	25	67.92 ± 8.56	234.37 ± 25.05	67.07 ± 2.21	.38
F10	40	50	50	65.18 ± 4.66	354.83 ± 48.81	65.64 ± 1.44	.51
F11	60	100	50	61.07 ± 4.53	440.24 ± 34.73	60.87 ± 1.95	.39
F12	40	150	15	65.94 ± 5.66	329.25 ± 28.78	70.59 ± 2.53	.49
F13	60	100	15	38.27 ± 2.01	391.11 ± 13.81	68.69 ± 3.69	.52
F14	20	100	50	68.35 ± 7.97	421.21 ± 28.18	70.65 ± 4.29	.49
F15	60	150	25	40.29 ± 5.28	466.36 ± 29.59	55.18 ± 2.61	.56

TBN: terbutaline sulfate; NVS: novasomes; EE%: entrapment efficiency percent; PS: particle size; Q_8h_: accumulative % release after 8 h; PDI: polydispersity index.

Drug concentration is constant (10 mg).

Data are mean values (*n* = 3) ± SD.

a Indicates the center point of the design.

### Preparation of TBN-NVS

For the preparation of TBN-NVS, the conventional thin-film hydration method was employed (Abd-Elal et al., 2016). In a 10 ml (1:1 v/v) chloroform/methanol organic solvent mixture, accurately weighed amounts of cholesterol, Span 60 and stearic acid were dissolved in round bottom flask. The organic solvents were slowly evaporated at 40 °C for 15 min under vacuum using a Stuart rotary evaporator (model RE300, Wolf Laboratories, North Yorkshire, UK) fixed with a Stuart vacuum pump (model RE3022C, Wolf Laboratories, North Yorkshire, UK). The evaporation process was terminated when a dry thin transparent film was obtained on the walls of the flask. Afterwards, the produced film was placed under vacuum in a desiccator for 2 h to ensure entire elimination of the traces of the organic solvents. Then, the resultant film was hydrated by utilizing phosphate buffer saline (PBS) pH 7.4 (10 ml) holding 10 mg TBN. The hydration process was maintained for 1 h at 60 °C with continuous rotation at 100 rpm for the formation of NVS. Sonication was carried out in a Sonix TV bath sonicator (model ss-series, North Charleston, SC) for 10 min for the purpose of particle size reduction. The assembled nanosuspensions of TBN-NVS were refrigerated overnight at 4 °C until further characterization.

## Characterization of TBN-NVS

### Entrapment efficiency percent of TBN (EE%)

Indirect estimation of the EE% was carried out after separating the novasomal suspension by subtracting free drug remained in the aqueous milieu from the whole drug integrated in the formulation. Suspension separation was achieved by centrifugation (Abou-Taleb et al., 2018) for 1 h using a SIGMA cooling centrifuge (model 3–30 K, Steinheim Germany) at rotation speed 14,000 rpm and temperature 4 °C. The supernatant was detached, diluted and then placed in a Jasco UV spectrophotometer (model V-530, Tokyo, Japan) for determination of the concentration of free non-entrapped TBN at 277 nm (maximum absorbance). Prior to the assay, a calibration curve in PBS pH 7.4 (R^2^, .984) was constructed over the range 20 to 120 µg/ml. The EE% was determined using the next equation:

(1) $$\mathrm{EE}\%=\frac{\left(\text{Total amount of TBN}-\text{free TBN amount}\right)}{\text{Total amount of TBN}}\;\times \;100$$

### TBN-NVS particle size (PS), ζ potential and polydispersity index (PDI)

The assembled TBN-NVS formulations were characterized for their mean PS, ζ potential and PDI utilizing a Malvern dynamic light scattering (DLS) Zetasizer ZS Nano 7.11 (Malvern, UK). Prior to the evaluation, samples of the novasomal preparations were appropriately mixed with double distilled water (Aboud et al., 2018). The assay was triplicated and mean values were reported.

### *In vitro* release behavior

The TBN-NVS formulations were characterized for their *in vitro* release behavior by using a locally fabricated Franz diffusion cell with an effective diffusion area of 5 cm^2^ and 10 cm length. In the donor chamber of the cell, a definite volume of TBN-NVS suspension (equivalent to 3 mg TBN) was introduced. Meanwhile, a 15 ml of PBS pH 7.4 was taken in the receptor compartment and kept at 37 ± .5 °C under continuous stirring at 50 rpm for 8 h (Jinturkar et al., 2012). Separation of the donor compartment from the receptor one was accomplished using a 12,000 Da molecular weight cut off cellulosic dialysis membrane. The cellulose membrane was initially dripped in PBS pH 7.4 and later it was fitted on the lower end of the diffusion cell to start the experiment. A 1 ml sample was withdrawn from the sampling port at .5, 1, 2, 3, 4, 5, 6, 7 and 8 h. The withdrawn sample was immediately replenished with a fresh milieu to ascertain a fixed volume. A .45 μm Millipore filter was used for filtration of the samples before they were subjected to spectrophotometric analysis at 277 nm using PBS (pH 7.4) as blank. After executing the *in vitro* release experimentations in a triplicate, the attained data were examined for determining the drug release kinetics from the various formulations. The magnitude of the coefficients of determination (R^2^) was employed for defining the most appropriate mathematical order.

### Formulation optimization

It is commonly to employ Design-Expert® software in order to statistically examine the impact of different formulation variables on the attributes of TBN-NVS to elect the optimal formulation reliant on the desirability index, which is preferred to be near one (Ahad et al., 2012). The optimized formulation of TBN-NVS was selected by applying constraints on (*Y_1_*: EE%) and (*Y_3_*: Q_8h_%) to reach their maximum values and on (*Y_2_*: PS) to attain its minimum value. The recommended TBN-NVS formulation was then fabricated and assessed (*n* = 3) in order to compare the formulation’s actual and software-predicted characteristics.

### Transmission electron microscopy (TEM)

To display the morphology of the optimized TBN-NVS formulation, the prepared nanovesicles were exposed to Jeol TEM (model JEM-1400, Tokyo, Japan). To perform TEM observations, one drop from the vesicular suspension was diluted, deposited on a carbon-coated grid and allowed to dry to be adsorbed into the carbon film. The sample was then treated with phosphotungstic acid 1% w/v as a negative stain. The grid was mounted in the instrument and soft imaging viewer software was run to take various photographs of the sample at different magnifications from diverse angles (Khallaf et al., 2020).

### Stability study of TBN-NVS

The optimized formulation of TBN-NVS was stowed in a glass vial at refrigeration temperature (4 ± 1 °C) for three months to assess its stability. The properties of the optimized TBN-NVS formulation were monitored at certain intervals (0, 30, 60 and 90 days). At the pre-defined day intervals, samples of the stored TBN-NVS formulation were aspirated and then evaluated for their physical appearance. Moreover, the mean values ± SD of 3 measurements of EE%, PS and ζ potential were computed and reported (Nasr et al., 2008; Khallaf et al., 2020).

### Aerodynamic particle size characterization

The frequently utilized instruments for determination of the extent and deposition patterns of nano-cargos administered through the pulmonic route are Anderson Cascade impactors (ACIs) (Nahar et al., 2013). ACI consists of eight stages and can provide valuable evidence on the size and pattern of deposition of particulate drug carriers in the respiratory system based on aerodynamic diameter (Mohammed et al., 2012). Once the aerosol stream passes through a certain collection plate, larger particles with adequate inertia cling to the plate, whilst comparatively smaller particles with inadequate inertia are transferred to the next impaction stage by the air stream (Mitchell & Nagel, 2003). Employing Copley inhaler testing data analysis, it is prudent to determine the fine particle dose (FPD) defined as the amount of particles with a size less than 5 µm, the fine particle fraction percent (FPF) correspondent to the percentage of the total amount deposited into the throat and stages of the cascade impactor and the mass median aerodynamic distribution (MMAD) representing the aerodynamic diameter at which half of the aerosolized drug mass is less than the stated diameter.

ACI was utilized to detect the particulate droplet size distribution of the emitted drug after tight insertion to the endotracheal tube without a cuff, size 5.5 system at ambient temperature. The dose of TBN-NVS suspension emitted to ACI was .2 mg. The endotracheal tube was tightly fitted into the ACI apparatus induction port on one side using an airtight seal and a syringe containing the drug was connected to the other end. The flow rate was adjusted using an automated digital flow meter to be at 28.3 l/min and it was provided through the ACI set *via* a vacuum pump. Afterwards, contents of each plate were washed with a definite volume of acetonitrile and assayed for the respective drug.

### Total emitted dose

The total emitted dosage described as the entire amount of medication ejected from the mouthpiece and expressed based on the nominal emitted dosage that represents the initial amount of medication introduced in the device (Salem et al., 2016). The medication delivery mechanism was constructed in such manner analogous to that of a rat receiving the TBN-NVS suspension *via* endotracheal tube. The flow rate was tuned to 28.3 l/min at ambient temperature and the settings were adjusted to imitate the breathing pattern in a healthy rat. An electrostatic filter pad bounded in a filter holder was attached next to the endotracheal tube. All aerosol produced during the emission of the dose from the syringe would be entrained on this filter and hence, the amount of the total inhaled dose can be predicted. The drug deposited on each filter was retrieved after complete immersion of the filter pad in acetonitrile followed by 3 min sonication. Thereafter, a vacuum was applied across the filter to assure that the total dose released by the delivery mechanism was captured (Abdelrahim et al., 2010; Hassan et al., 2016). A 100 µl of 2 mg/ml TBN-NVS nanosuspension was emitted to the endotracheal tube and the experimentation was replicated to obtain six consecutive measurements. Acetonitrile was used to rinse each filter, endotracheal tube and inside the tubing for retrieving all amounts of the deposited drug.

The quantity of TBN in the samples was estimated using a modulated liquid chromatography–mass spectrometry method (LC-MS/MS) (Domínguez-Romero et al., 2013). The LC system (Shimadzu Controller CBM20Alite, Japan) was equipped with LC-MS/MS detector (triple quadrupole), a quaternary pump (LC20AD) and autosampler (SIL20A). Positive ion mode separation and quantitation were accomplished utilizing a mass spectrometer (AB Sciex API-4000). The mobile phase was prepared daily from a 80:20% v/v acetonitrile/.1% formic acid mixture and the flow rate was set at 1 ml/min. The nebulizer gas pressure was 35 psi and the voltage of ion spray was 3.6 kV.

### *In vivo* studies

The protocol of the *in vivo* studies was approved by our institutional Animal Ethics Committee of Beni-Suef University in agreement with the guidelines of the National Institutes of Health Guide for Care and Use of Laboratory Animals (approval code: REC-A-PhBSU-20016). Male Wistar rats (250–300 g) were involved in these studies. Animals were housed in wide mesh wire cages with unrestricted access to food and water on a 12/12 h light/dark cycle and humidity-controlled rooms within the animal house (temperature 25 ± 2 °C). Ketamine (12.5 mg/kg) and xylazine (1.5 mg/kg) were co-injected intraperitoneally into the animals to induce anesthesia (Hamzawy et al., 2017). Microsprayer® IA-1C i.t instillation system (Penn-Century, Philadelphia, PA) was used for the administration of the optimized formulation of TBN-NVS suspension (Bivas-Benita et al., 2005).

### *In vivo* histopathological analysis

To detect ultrastructural changes in the lung tissue upon the introduction of i.t TBN-NVS suspension, an *in vivo* histopathological analysis was executed. Eight rats were involved in this study and they were arbitrarily distributed into two equal sized groups (*n* = 4). The animals in group A served as the control, while those in group B were the treated group that received an i.t suspension of the optimized TBN-NVS for 14 days. At the end of the experimentation, the rats were euthanized and their lungs were collected and fixed in formalin solution until examination. For the histopathological inspection, lungs were submerged in paraffin wax blocks, maintained at 56 °C for 24 h and then cut into a series of 5 µm thick sections utilizing a microtome. Afterwards, a light microscope was employed to examine the cut sections after staining with hematoxylin and eosin (H & E) according to the protocol previously reported by Bancroft and Gamble (2008). At different magnifications, the sections were photographed using LEICA digital camera system (model DFC290HD, Heerbrugg, Switzerland) attached to a light microscope.

### Pharmacokinetic study

Twelve rats were utilized in the pharmacokinetic study and they were divided into three groups each comprising four animals. Group A received 1 ml of an oral TBN solution in PBS pH 7.4 (200 μg/ml), while animals in groups B and C received 100 μl of i.t administered TBN solution in PBS pH 7.4 and the optimized TBN-NVS suspension, respectively, equivalent to 200 μg/100 μl after intraperitoneal anesthesia. The doses that administered *via* the i.t route were followed by 50 μl of .9% saline to wash the syringe and Microsprayer® tubing (Joshi & Misra, 1999). Samples of blood (500 µl) were collected at .5, 1, 2, 4, 8 and 24 h from the retro-orbital venous plexus of each rat into heparinized tubes. Cooling centrifugation at 3000 rpm for 15 min was employed to isolate plasma from the whole blood of the collected samples. The resultant supernatant was preserved at −20 °C for further analysis. The LC-MS/MS system, as described before, was used for analysis of plasma samples and Shimadzu Controller Version Analyst 1.6 was used for calculation of drug concentration.

### Preparation of samples for analysis

An aliquot of .5 ml of separated plasma was mixed with 4 ml of tertiary butyl methyl ether and vortexed for 5 min. The denatured protein precipitate was then extracted by cooling centrifugation for 10 min at 4 °C. The supernatant was further centrifuged under vacuum for 15 min at 4000 rpm for evaporation of the organic layer. The resulting residue was dissolved in .25 ml of mobile phase and then injected into the LC column.

## Data analysis

The pharmacokinetic parameters were computed by non-compartmental analysis using WinNonlin standard edition software (Version 1.5, Scientific Consulting Inc., Pharsight Corp., Cary, NC, USA). *C_max_* in ng/ml was estimated as the greatest observable concentration throughout the study duration and *T_max_* (h) is the time needed to reach the *C_max_*. The area under the plasma concentration − time curve up to the last measured time point (*AUC_0 − 24_*, ng h/ml) was calculated by the trapezoidal rule. The residual area was obtained by dividing the concentration at the last recorded time point by the elimination rate constant and then, was used for the calculation of *AUC_0_****_–∞_*** (ng h/ml). The percentage relative bioavailability (*F_rel_*) for both i.t formulations, with respect to oral solution as a standard, can be assessed as follows:

(2)$$F_{\mathrm{rel}}=\frac{{\mathrm{AUC}}_{0-\infty \;}(i.t\;\mathrm{formulation})}{{\mathrm{AUC}}_{0-\infty }(\mathrm{oral}\;\mathrm{solution})}\times 100\;(2)$$

## Statistical analysis

The results of the pharmacokinetic parameters were analyzed using one-way ANOVA with subsequent multiple comparisons using Tukey post-hoc. The computer program SPSS 22 (Chicago, IL) was employed to process all computations. Data were expressed as mean ± SD and the level of significance was set at .05.

## Results and discussion

### Preparation of TBN-NVS

Preliminary studies were substantial for the selection of the most appropriate conditions for preparing TBN-NVS formulations. Our main target was to incorporate the water-soluble drug TBN in the novasomal suspension, thus, the EE% was a critical parameter in the design of the TBN-NVS formulations. Actually, several surfactants were tested and the highest values of EE% were observed when Span 60 was used in a combination with cholesterol. Also, different types of free fatty acids were inspected and stearic acid exhibited the utmost values of EE% and acceptable ranges of PS. Therefore, these results were encouraging to select the three factors: cholesterol (*X_1_*), Span 60 (*X_2_*) and stearic acid (*X_3_*) concentrations to estimate their effects at different levels in order to optimize the TBN-NVS based on Box-Behnken statistical design. The film hydration technique was adopted to prepare TBN-NVS and 10 ml of 1:1 chloroform/methanol mixture was utilized to produce a clear and continuous thin film.

### Characterization of TBN-NVS

**Figure 1.**
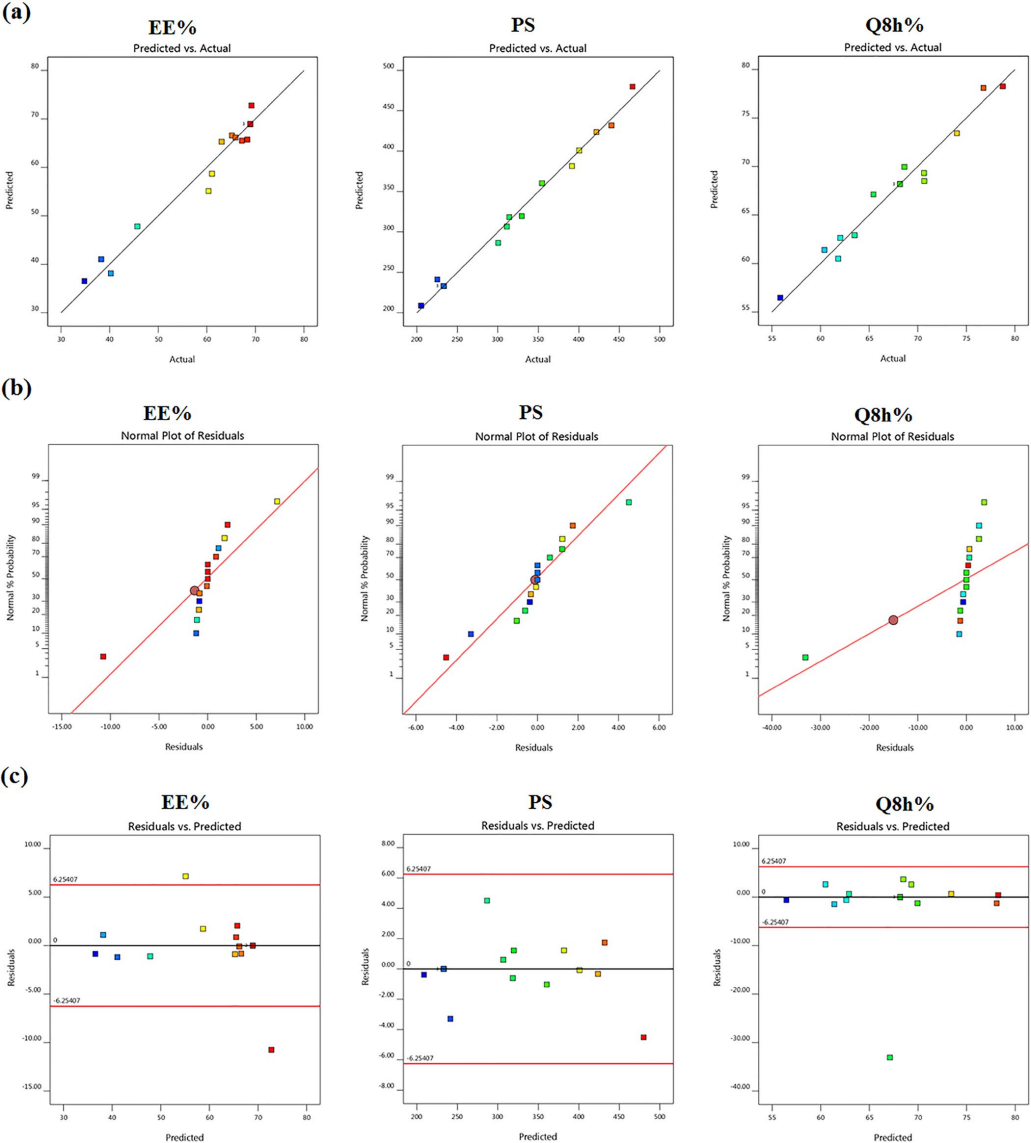
Model diagnostic plots of the three independent variables (a) linear correlation plots between actual and predicted values for various responses of TBN-NVS, (b) normal quantile-quantile plots of residual errors and (c) plot of residual error vs. model predicted responses.

A total of 15 formulations were nominated by using the Design Expert® software and the observed responses are given in Table 2. It can be manifested that all the three independent variables, the concentrations of cholesterol (*X_1_*), Span 60 (*X_2_*) and stearic acid (*X_3_*) had interactive effects on the three responses (*Y_1_*: EE%), (*Y_2_*: PS) and (*Y_3_*: Q_8h_%). Figure 1(a)–(c) illustrates the model diagnostic plots of the three causal factors which elucidate that the models adequately suited the data, with residual error that is free from discernible patterns and almost normally distributed.

### Effect of formulation variables on EE%

**Figure 2.**
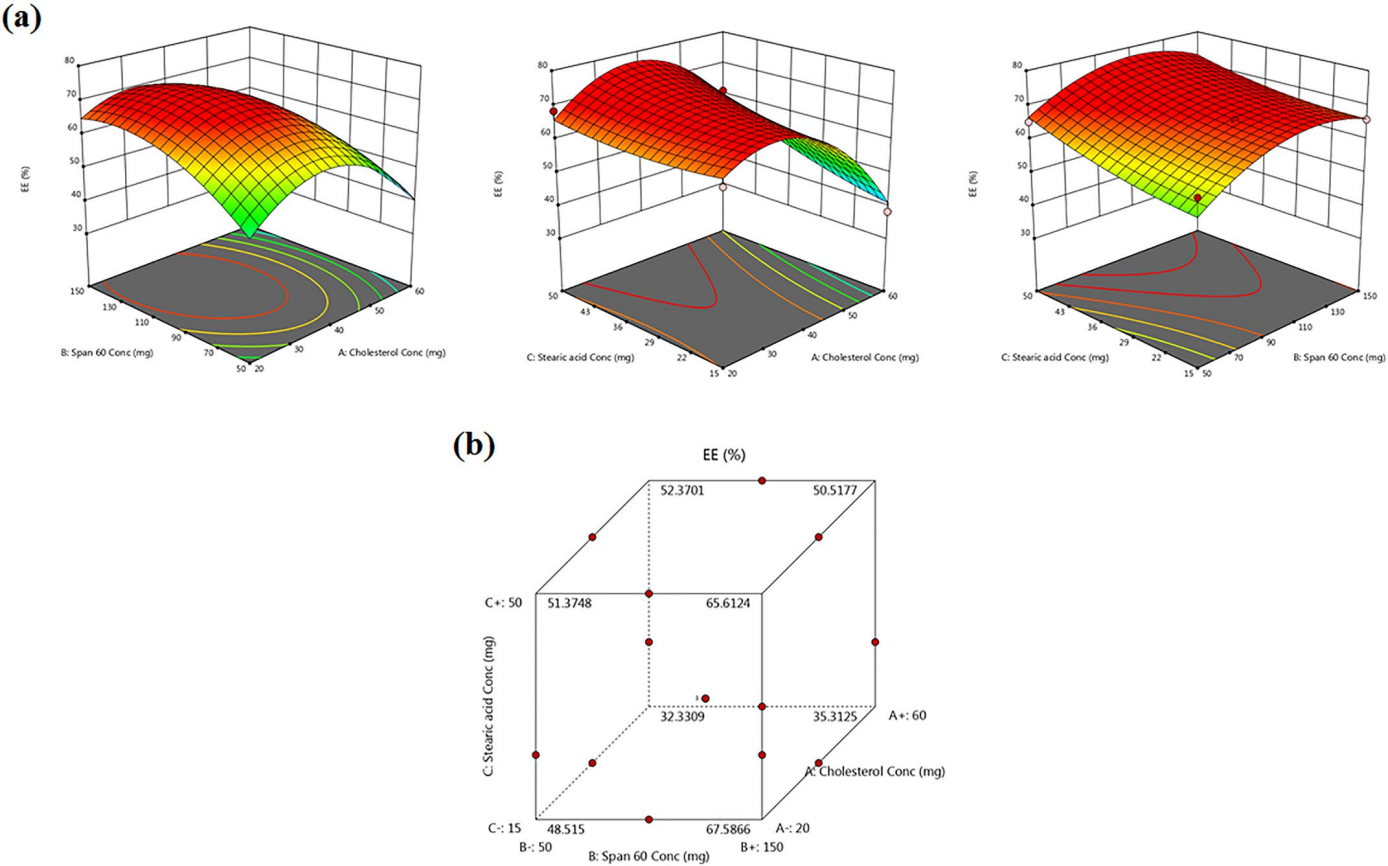
(a) Response 3D plots and (b) cube plot for the effect of cholesterol (*X_1_*), Span 60 (*X_2_*) and stearic acid (*X_3_*) concentrations on the entrapment efficiency percent (*Y_1_*).

EE% is the percentage fraction of the total drug loaded into the NVS calculated as a ratio from the original drug mass. EE% measurements of the total 15 formulations are presented in Table 2. Figure 2 displays the effects of the three causal factors on the EE% in the form of response surface and cube plots. The data clearly disclosed that TBN was successfully incorporated within the novasomal formulations with EE% varying between 34.87 ± 3.23 and 68.93 ± 6.04%. A quadratic model was deemed statistically fit by ANOVA analysis of TBN-NVS recorded EE% data. The yielded polynomial equation represented as coded independent variables is denoted as:

(3)$$EE\%=+70.50-7.81X_{1}+4.30X_{2}+4.51X_{3}-4.02X_{1}X_{2}+4.29X_{1}X_{3}-1.20X_{2}X_{3}-14.68X_{1}^{2}-7.22X_{2}^{2}+1.86X_{3}^{2}\;(3)$$

All the three independent variables have divulged a significant effect on the EE% (*p* = .004). Notably, there was a marked reduction in the EE% of TBN with gradual elevation of cholesterol content (*X_1_*) in the TBN-NVS formulations (*p* = .003), Figure 2. Al-mahallawi et al. (2014) shared similar findings upon assessing the properties of ciprofloxacin-laden nanotransfersomes for trans-tympanic delivery. The notion that upraised hydrophobicity triggered by cholesterol within the central region of the membrane bilayers might have provoked the expulsion of hydrophilic moieties such as TBN (Salem et al., 2018), especially when considering the bulky nature of cholesterol (Ali et al., 2010).

According to Figure 2 and Table 2, it can be observed that there was a synchronous upsurge in TBN EE% with further increments in the concentration of Span 60 (*X_2_*) (*p* = .034). This effect might be ascribed to the pronounced drug encapsulation of Span 60 conferred by its higher phase transition temperature (53 °C). Another hypothetical clarification could be interrelated to the lengthier saturated hydrocarbon chain (C14) of Span 60 which might donate robustly stable NVS bilayers with subsequent accentuated EE% (Aboud et al., 2016). Furthermore, Span 60 possesses a lower HLB value of 4.7 which renders it highly hydrophobic and confers a consolidated EE% (Yoshioka et al., 1994). Parallel observations were presented elsewhere (Khallaf et al., 2020) as olanzapine-based niosomes with higher Span 60 content possessed higher EE%.

Also, the positive coefficient of *X_3_* indicates that upraising the total concentration of stearic acid gave rise to a significant escalation in the EE% of the prepared TBN-NVS (*p* = .025). This observation might be correlated to the tight straight alkyl chain (C18) of stearic acid (Kanicky & Shah, 2002), which resulted in less permeable nanovesicles with a concomitant higher EE% (Abd-Elal et al., 2016).

### Effect of formulation variables on PS

**Figure 3.**
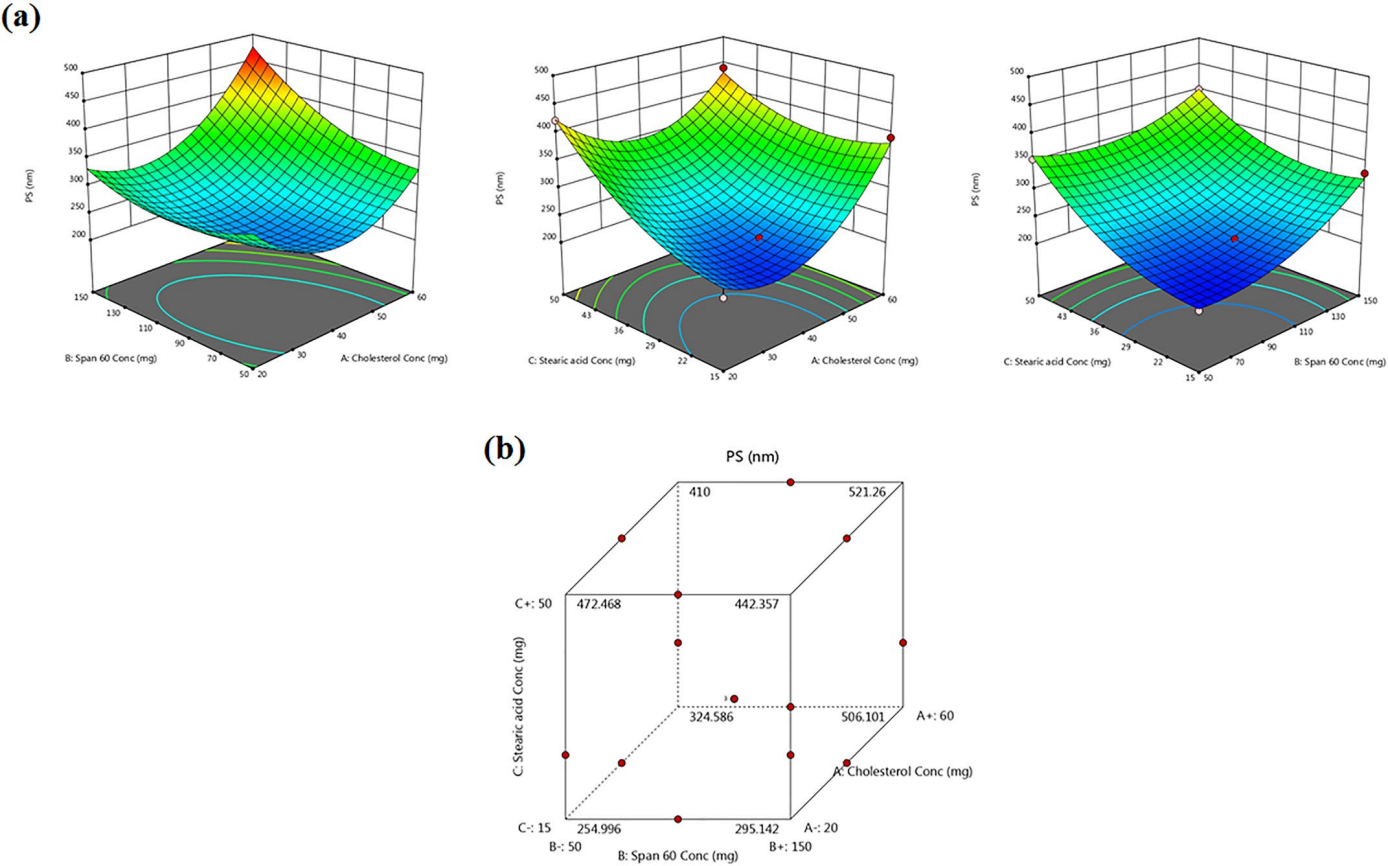
(a) Response 3D plots and (b) cube plot for the effect of cholesterol (*X_1_*), Span 60 (*X_2_*) and stearic acid (*X_3_*) concentrations on the particle size (*Y_2_*).

The PS and shape parameters play a vital role in the lung deposition of nano-cargos. The average vesicular sizes of diverse TBN-NVS are depicted in Table 2 and graphically illustrated in Figure 3. All the NVS formulations exhibited a nano-size range between 205.94 ± 6.82 and 466.36 ± 29.59 nm which is appropriate for superior cellular uptake and competent drug delivery into the lung (Jinturkar et al., 2012) due to increased diffusion mobility (Patlolla et al., 2010). The ANOVA test demonstrated that the recorded PS data comply with a quadratic model. The equation for TBN-NVS PS in terms of coded independent variables is:

(4)$$PS=+251.17+37.12X_{1}+37.85X_{2}+58.15X_{3}+35.34X_{1}X_{2}-33.01X_{1}X_{3}-17.56X_{2}X_{3}+80.98X_{1}^{2}+33.78X_{2}^{2}+37.42X_{3}^{2}\;(4)$$

The equation elicits positive significant effects for all the three independent variables on the monitored response (*Y_2_*) (*p* = .0002). For cholesterol concentration (*X_1_*), the observed significant repercussion on the PS (*p* = .0008) is in line with Fetih (2016) who fabricated fluconazole-laded niosomal gels for ocular delivery. The bulky structure of cholesterol was thought to increase the distance between NVS bilayers, hence providing a larger diameter size of TBN-NVS (Ali et al., 2010; Al-mahallawi et al., 2014).

As well, the PS was found to significantly increase with the upsurge of Span 60 concentration (*X_2_*) (*p* = .0008). These findings are coordinated with EE% results, where integrating higher content of Span 60 triggered greater EE% of TBN consequently, the whole PS of TBN-NVS was expanded. This finding is harmonious with that of El Menshawe et al. (2019) who claimed the positive impact of Span 60 on the vesicular size of fluvastatin sodium-based spanlastics.

Regarding stearic acid concentration (*X_3_*), the 3D-graph and cube plot as clarified in Figure 3 elucidated significant growth of the novasomal vesicles as the amount of stearic acid continued to pile up in the formulation (*p* < .0001). The enlargement could be referred to the influence of raised viscosity acquired as the quantity of stearic acid was increased (Araujo et al., 2010; Chen et al., 2010). Such findings coincide with those narrated by Baig et al. (2016) upon formulation of levofloxacin-loaded solid lipid nanoparticles.

The PDI was used to investigate the width of the PS distribution and the total homogeneity of the particulate size within the nanodispersion. A large PDI value indicates a heterogeneous distribution of the vesicles while a small one reflects a homogenous monodispersed size distribution (Mahmoud et al., 2017). The PDI of all the TBN-NVS formulations oscillated between .31 and .59 revealing a polydispersed system having neither a very narrow (PDI < .05) nor a very broad (PDI > .7) size distribution, Table 2.

### Effect of formulation variables on drug release

**Figure 4.**
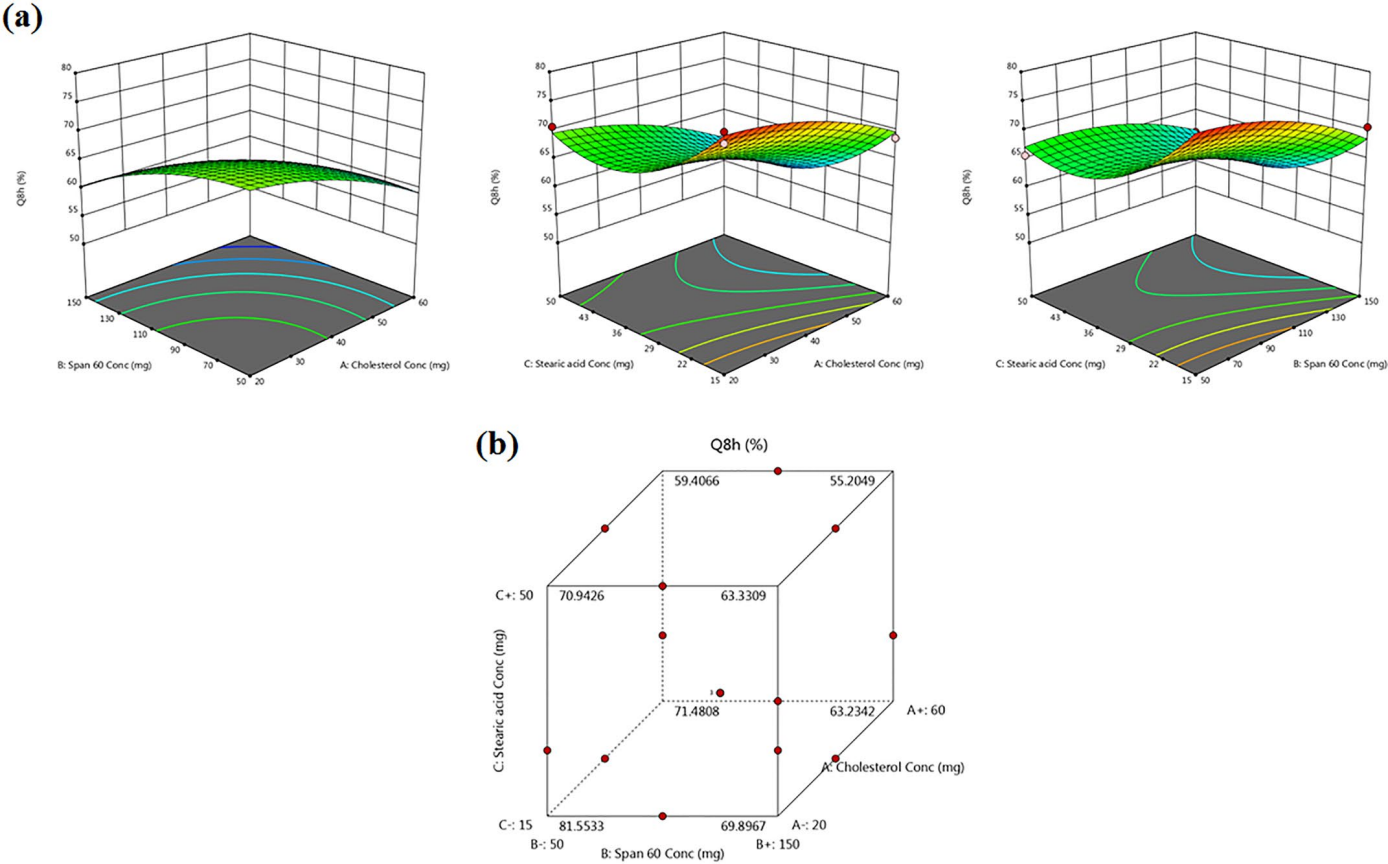
(a) Response 3D plots and (b) cube plot for the effect of cholesterol (*X_1_*), Span 60 (*X_2_*) and stearic acid (*X_3_*) concentrations on the accumulative % drug release after 8 h (*Y_3_*).

Table 2 records the results of *in vitro* TBN release from the different TBN-NVS formulations and Figure 4 reveals the joint influence of the independent formulation variables on Q_8h_% of the drug in 3D-graph and cube plots. The drug release profiles of TBN-NVS were biphasic, much slower and more sustained than that of TBN solution. This behavior is largely consistent with the remarkable reservoir impact of various vesicular nano-cargos triggering an extended release of the laden drugs (Aboud et al., 2016; Mahmoud et al., 2017; Aboud et al., 2018; Salem et al., 2022). The first release phase was characterized by an initial burst effect (approximately 40% drug released in the first 2 h) resulting from the free drug present in the nanosuspension and the hydrophilic nature of TBN (Khalil et al., 2019). The second phase was a slower sustained release due to the efflux of the entrapped drug through the lipid bilayers of the NVS. The data also showed that the TBN-NVS formulations released from 55.18 ± 2.61 to 78.70 ± 1.34% of their drug content after 8 h, Table 2. Like EE% and PS, the quadratic model was found to be significant and adequate for the release data based on ANOVA analysis of the Box-Benkhen design. The joint influence of the independent formulation variables on Q_8h_% of TBN was described by the equation:

(5)$$Q_{8h}\%=+65.04-4.55X_{1}-3.96X_{2}-4.66X_{3}+.8525X_{1}X_{2}-.3659X_{1}X_{3}+1.01X_{2}X_{3}-1.97X_{1}^{2}-2.40X_{2}^{2}+6.21X_{3}^{2}\;(5)$$

By further inspection of the results, it was obvious that cholesterol (*X_1_*) content had significant antagonistic consequences on the Q_8h_% of TBN-NVS (*p* < .05). Indeed, the presence of cholesterol aroused good stability for the vesicles wherein a more rigid and less leaky membrane was formed (Deniz et al., 2010). Such firm membrane resulted in diminution of TBN outflow from the TBN-NVS, accordingly the drug release was retarded (Hathout et al., 2007).

Similar to cholesterol, the results indicated an unfavorable depressive effect of Span 60 concentration (*X_2_*) on the Q_8h_% (*p* = .002). Presumably, the amalgamation of surfactant molecules in the formulation confers favorable drug release behavior owing to the induction of significant alterations in the bilayer membrane of the vesicles. These alterations encompass higher elasticity, deformability and fluidity (Salem et al., 2019). Such discrepancy could be argued on the basis of dissimilarities in molecular ordering primed by surfactants, provoking variation in the lipidic bilayers fluidity and overall vesicular deformability. Since our release experimentation was executed at 37 °C, diminished rates of TBN release from the NVS could be ascribable to the elevated transition temperature of Span 60, as mentioned earlier, practically providing them in a rigorously ordered gelled assembly.

Likewise, the negative relationship between the Q_8h_% of TBN and the incorporated amount of stearic acid (*X_3_*) could be interpreted in the light of the alkyl chain of the stearic acid. The alkyl carbon chain of the stearic acid is long, tight and straight (Abd-Elal et al., 2016) which resulted in less leaky nanovesicles. Hence, retarded TBN release from the TBN-NVS containing high concentration of stearic acid is an expected attitude.

The *in vitro* release data for most TBN-NVS formulations obeyed the kinetic model of Higuchi over the zero-order and first-order models as found by the determination coefficient values (data not shown). This finding could imply that the drug was released *via* diffusion, i.e. sluggish and prolonged release of the drug occurred through its diffusion from the matrix of the NVS system, as postulated by Higuchi.

### Formulation optimization and analysis of Box-Behnken design

Box-Behnken design was adopted for designing, analyzing and optimizing the TBN-NVS system. Such design was proposed as it entails a limited number of experiments for making an optimization (Goyal et al., 2015). Box-Behnken statistical analysis disclosed the mathematical model aptitude to investigate the significant effect of the causal factors on the monitored responses. Adequate precision is a measure of the signal to noise ratio which is required to be more than 4 to ascertain that the model is reliable enough to explore the space of the design (Abdelbary & AbouGhaly, 2015). All responses had shown a ratio greater than 4 signifying an appropriate model as profiled in Table 3. Another estimate of how good the model anticipates a response value is the predicted R^2^ (Kaushik et al., 2006). The adjusted and predicted R^2^ should be within nearly .20 of each other to be in a sensible agreement (Annadurai et al., 2008). Worthwhile, the adjusted R^2^ for all three responses was in close conformity with those of the predicated ones. The optimized formulation of TBN-NVS was picked based on the specified criteria of accomplishing maximum values of EE% and Q_8h_%, and minimum values of PS adopting the numerical point prediction optimization approach of the Design Expert software®. Design Expert® recommended the optimized formulation of TBN-NVS to be prepared with an overall desirability of .972. The composition of the optimized TBN-NVS formulation was 30.77 mg cholesterol, 90.72 mg Span 60 and 15 mg stearic acid. The optimal TBN-NVS formulation was assembled in triplicate and the dependent responses were estimated. The optimal formulation exhibited EE% of 67.48 ± 5.23%, vesicle size of 223.89 ± 22.41 nm and Q_8h_% of 80.11 ± 7.25%. As compiled in Table 4, the optimal formulation observed and model expected response values were strongly comparable signaling an extremely low prediction error percentage oscillating between −5.21 and 6.55% for the various responses. This finding stresses the suitability and fitness of the proposed mathematical models for exploring the response space of the dependent variables. Accordingly, this formulation was chosen for additional studies.

**Table 3.** Results of regression analysis for responses *Y_1_* (EE%), *Y_2_* (PS) and *Y_3_* (Q_8h_%).

Response	Model	Adequate precision	R^2^	Adjusted R^2^	Predicted R^2^	SD	CV%	*P* value
*Y_1_*: EE%	Quadratic	10.39	.9627	.8955	.8859	4.06	6.87	.0046
*Y_2_*: PS	Quadratic	23.58	.9905	.9733	.8494	14.08	4.35	.0002
*Y_3_*: Q_8h_%	Quadratic	16.58	.9656	.9316	.9193	1.67	2.48	.0016

EE%: entrapment efficiency percent; PS: particle size; Q_8h_: accumulative % release after 8 h; R^2^: coefficient of determination; SD: standard deviation; CV: coefficient of variation.

**Table 4.** TBN-NVS optimal formulation composition with the laboratory measured and model predicted characteristics.

Factor	Optimal value	Response	Measured value	Predicted value	% Prediction error^a^
*X_1_*: Cholesterol (mg)	30.77	*Y_1_*: EE (%)	67.48 ± 5.23	71.00	−5.21
*X_2_*: Span 60 (mg)	90.72	*Y_2_*: PS (nm)	223.89 ± 22.41	209.23	6.55
*X_3_*: Stearic acid (mg)	15.00	*Y_3_*: Q_8h_ (%)	80.11 ± 7.25	78.33	2.22

TBN: terbutaline sulfate; NVS: novasomes.

a % Prediction error = (Measured − Predicted/Measured*100).

### Transmission electron microscopy (TEM)

**Figure 5.**
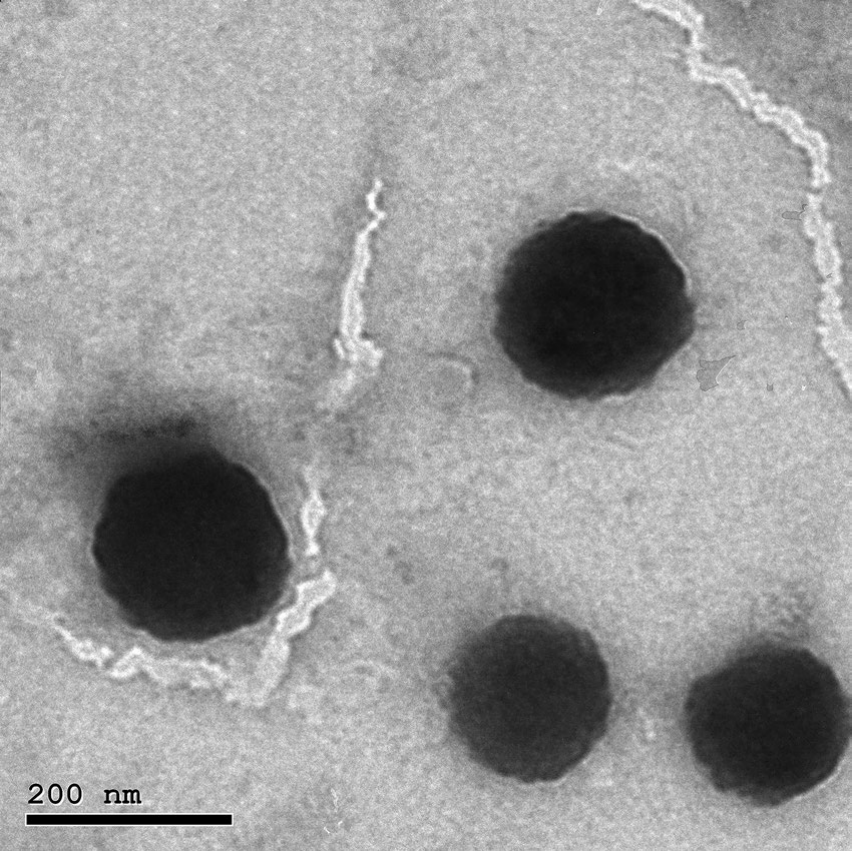
Transmission electron micrograph of the optimized TBN-NVS formulation.

TEM was originally used to investigate the morphology of the optimized TBN-NVS formulation. The TEM image represented a homogeneous size distribution of the lipidic unilamellar nanovesicles with a roughly spherical shape as manifested in Figure 5. Additionally, the vesicles were well dispersed and well separated. Moreover, the size analysis obtained by the TEM micrographs confirmed the nanosized range which was congruent with that obtained by DLS.

### Stability study of the TBN-NVS

**Figure 6.**
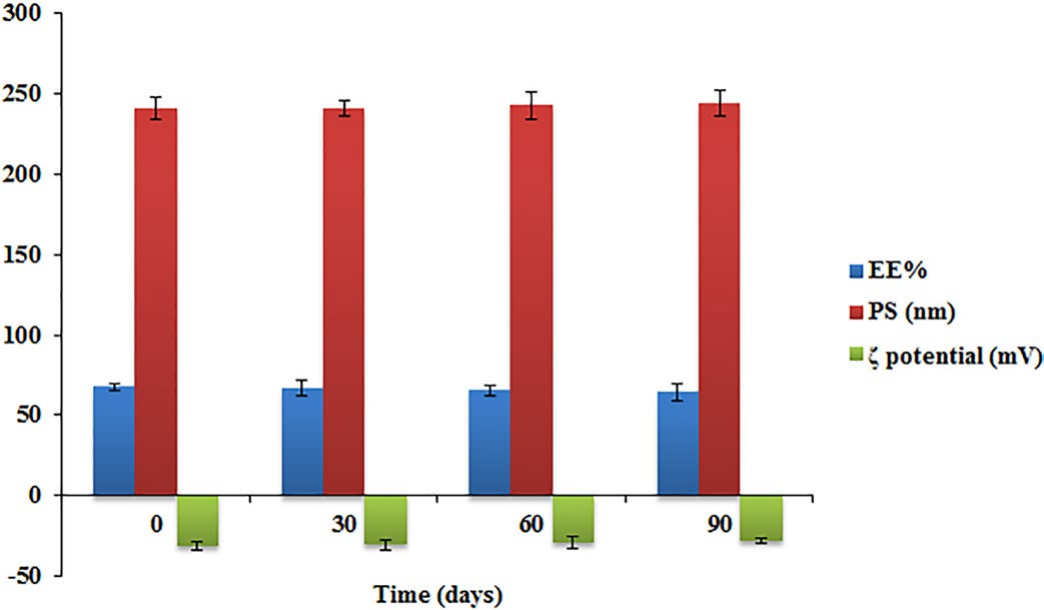
Effect of storage on the EE%, particle size and ζ potential of the optimized TBN-NVS formulation.

The physical stability of the optimized TBN-NVS formulation was assessed for the values of EE%, PS and ζ potential after storage for a 3-month period at 4 °C. No aggregation or abnormality was noted during the period of storage. The data graphically demonstrated in Figure 6 revealed that the length of storage had a little insignificant influence on the EE%, PS and ζ potential of the formulation (*p* > .05). The ζ potential was determined for the optimal formulation as it is regarded as a beneficial factor in mapping stability profile of drug delivery systems with colloidal nature. It represents the quantity of the electrical charge on the surface of the vesicles. High ζ potential values denote great repulsion phenomenon between nanovesicles, hence leading to a more stable colloidal dispersion (Aboud et al., 2020). The optimized TBN-NVS formulation exhibited a highly negative ζ potential (−31.17 ± 2.58 mV) indicating quality of dispersion. The negative charge on the surface of NVS might be owed to the presence of stearic acid that increased NVS stability in the aqueous phase (Salem et al., 2016).

### Aerodynamic particle size characterization

*In vitro* methods confer a quality assurance procedure for an inhaler device to determine the quality of the inhalable released product. Additionally, they are frequently extrapolated to provide an estimate of *in vivo* formulation deposition into the lungs. Furthermore, the TBN-NVS particulate dimension is critical for determination of the destination, depth of penetration and accumulation within the lung mucosal membranes (Kuzmov & Minko, 2015). The results revealed that the majority of the emitted TBN-NVS were detected on ACI stages 3–5. Relatively, very minute amounts were collected on stages 6 and 7. The aerodynamic traits of the tested formulation are listed in Table 5. The aerosol from the endotracheal tube had MMAD of 3.30 ± .06 μm and FPF of 86.44 ± 6.32% referring to a high propensity for deep deposition of the optimized TBN-NVS into the lung tissue. Therefore, these results implied that the tailored TBN-NVS would have a powerful pharmacological efficiency in lung diseases.

**Table 5.** Cascade impaction results of TBN-NVS.

Aerodynamic parameter	Value
TED (µg)	144.64 ± 12.81
TED as percentage of nominal dose (%)	72.32 ± 5.54
FPD (µg)	125.03 ± 6.71
FPF (%)	86.44 ± 6.32
MMAD (µg)	3.30 ± .06

TBN: terbutaline sulfate; NVS: novasomes; TED: total emitted dose; FPD: fine particle dose; FPF: fine particle fraction; MMAD: mass median aerodynamic diameter.

Results are means ± SD (*n* = 6).

## *In vivo* studies

### Histopathological examination

**Figure 7.**
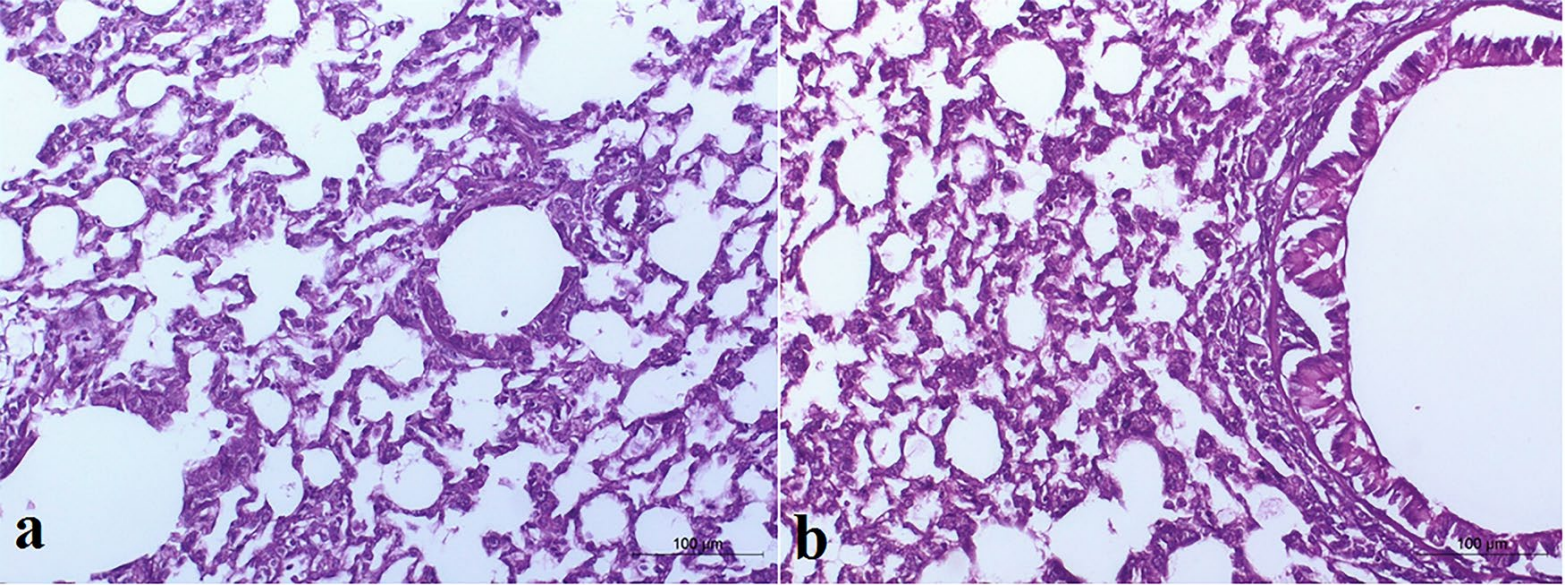
Light photomicrographs showing histopathological sections of (a) control untreated rat lung and (b) rat lung received an intratracheal suspension of TBN-NVS formulation (200X H & E).

Histopathological investigation of the lung tissues of the rats intratracheally-inhaled with the optimal TBN-NVS in comparison to normal lung tissues was implemented for detection of any acute toxicity of the nanovesicular dispersion. As the inhalation procedure might trigger heterogeneous distribution of the drug, both the right and left lungs were separately inspected. The control group was devoid of any signs of peribronchial or perivascular neutrophil inflammation, edema or epithelial damage, Figure 7(a). As elucidated in Figure 7(b), no tissue inflammation or damage was distinguished over both left and right lungs of the animal treated with the i.t suspension of the optimized TBN-NVS formulation. Such observations run with that obtained by Levinsky et al. (1978) who declared safety and tolerability of TBN following a three-month investigation of inhalation-toxicity in squirrel monkeys. Additionally, the results demonstrated the absence of acute toxicity related to the nano-cargo components.

### Pharmacokinetic analysis

**Figure 8.**
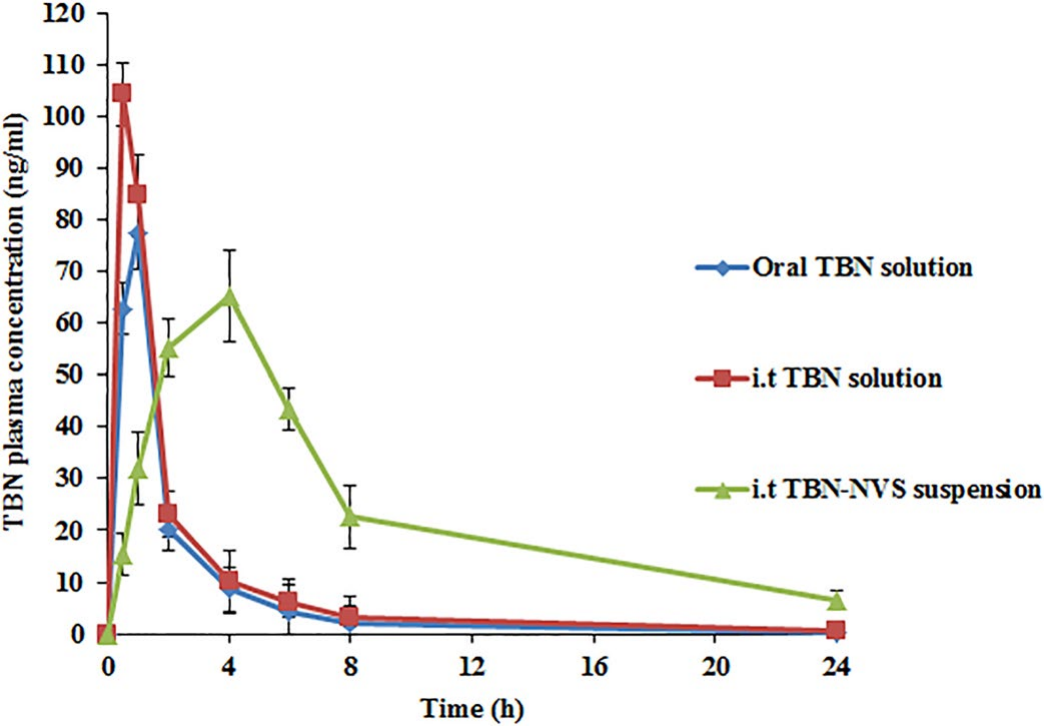
TBN plasma concentration time profiles after administration of oral TBN solution, i.t TBN solution and i.t TBN-NVS suspension.

TBN concentrations after administration of the optimized i.t TBN-NVS suspension, i.t TBN solution and also the oral TBN solution were quantified in the plasma of Wistar male rats. The time course of average plasma TBN concentration and the derived pharmacokinetic parameters associated with various formulations are revealed in Figure 8 and Table 6, respectively.

**Table 6.** The mean pharmacokinetic parameters of TBN in rat plasma following administration of oral TBN solution, i.t TBN solution and i.t TBN-NVS suspension.

Pharmacokinetic parameter	Formulation		
	Oral TBN solution	i.t TBN solution	i.t TBN-NVS suspension
C_max_ (ng/ml)	77.41 ± 5.33	104.27 ± 10.99^a^	65.22 ± 4.21^a^ ^,b^
T_max_ (h)	1.00 ± .25	.50 ± .00^a^	4.00 ± .00^a^ ^,b^
K_e_ (h^−1^)	.21 ± .03	.13 ± .03^a^	.10 ± .004^b^
t_1/2_ (h)	3.30 ± .62	5.33 ± .37^a^	6.93 ± .10^a^ ^,b^
AUC_0–24_ (ng h/ml)	167.99 ± 7.20	218.93 ± 15.87^a^	588.84 ± 24.86^a^ ^,b^
AUC_0–∞_ (ng h/ml)	168.47 ± 11.11	223.41 ± 16.17^a^	655.24 ± 27.95^a^ ^,b^
F_rel_ (%)	–	132.61	388.93^b^

TBN: terbutaline sulfate; NVS: novasomes.

Data presented are mean ± SD, *n* = 4.

Using one-way ANOVA followed by Tukey’s post-hoc test.

a *p* < .05 vs. oral TBN solution.

b *p* < .05 vs. i.t TBN solution.

TBN is a typical water-soluble drug so when TBN solution was administered through oral or i.t routes, it was rapidly detected in plasma. The *T_max_* for oral TBN solution and i.t TBN solution were 1.00 ± .25 and .50 ± .00 h, respectively, but unfortunately, they were cleared from the circulation within less than 8 h. Controversially, the i.t suspension of the optimized TBN-NVS formulation reached its *T_max_* within 4 h and it could be detected in the plasma up to 24 h. The *AUC_0-∞_* and *C_max_* of TBN in plasma were 168.47 ± 11.11 ng h/ml and 77.41 ± 5.33 ng/ml, 223.41 ± 16.17 ng h/ml and 104.27 ± 10.99 ng/ml as well as 655.24 ± 27.95 ng h/ml and 65.22 ± 4.21 ng/ml following delivery of the TBN oral solution, TBN i.t solution and TBN-NVS i.t suspension, respectively. The i.t TBN-NVS suspension administration had a distinctively higher *AUC_0-∞_*value by nearly 3.9- and 2.9-fold (*p < .05*) compared to both oral and i.t TBN solutions, respectively. The i.t TBN-NVS suspension signaled a *t_1/2_* value of 6.93 ± .10 h, while the oral and i.t TBN solutions showed *t_1/2_* values of 3.30 ± .62 h and 5.33 ± .37 h, respectively. The sustained and slow absorption of TBN through i.t instillation of TBN-NVS suspension was elucidated by the retarded *T_max_* and prolonged *t_1/2_* suggesting the efficacy of the NVS reservoir to protect TBN from enzymatic degradation in the lung (Arafa & Ayoub, 2018). The *F_rel_* of TBN from the i.t TBN-NVS suspension was about 388.93% compared to the oral solution and it was 132.61% for the i.t TBN solution. It could be noted that i.t instillation of TBN either as a solution or as a nanosuspension showed a more significant enhancement in the pharmacokinetic parameters than that of the oral TBN solution over a reasonable period of time indicating that TBN pulmonary administration is capable of accomplishing a targeted effect. Overall, such snowballed pulmonic TBN pharmacokinetics following i.t administration of TBN-NVS could be accredited to diverse mechanisms: (i) biocompatibility and biodegradability of NVS constituents conferring superlative *in vivo* tolerability for mucosal membranes (Aboud et al., 2022); (ii) optimum MMAD and FPF of the tailored nano-cargo which enabled adequate drug delivery to the pulmonary epithelia, thus evading the physiological bio-barriers of the respiratory system and residing for longer time (Elkomy et al., 2021); (iii) smaller vesicular size of the optimal NVS formulation since it was claimed that nanocarriers of particulate size less than 500 nm elicit boosted drug deposition through entire lung tissues predominately owing to the accentuated diffusional mobility (Patlolla et al., 2010); (iv) breaking away from the intensive hepatic first-pass metabolism concomitant with oral administration of TBN; (v) prolonged circulation time pursuant to TBN loading into NVS which could mitigate the pulmonary enzymatic degradation of the drug and (vi) the permeation enhancing aptitude of the vesicular nano-cargo exerted by the combined impact of novasomal hydrophilic and hydrophobic moieties (Aboud et al., 2022).

## Conclusion

The hydrophilic β_2_ adrenoceptor agonist, TBN, was successfully encapsulated in the NVS core. The optimal TBN-NVS formulation displayed a nanoscale diameter size with adequate aerodynamic properties. The TBN-NVS formulation enabled TBN release over an extended period of time in a sustained manner. Considering physical stability, the TBN-NVS formulation demonstrated accentuated stability over the storage period. The pharmacokinetics results manifested that the pulmonary route is a promising surrogate to maintain therapeutic TBN efficacy, minimize clearance, maximize delivery and diminish local and systemic toxicity. Furthermore, the current outcomes display a high clinical treatment potential of NVS as a talented nanovector for the pulmonary delivery of TBN.

## Authors’ contributions

Conceptualization, H.M.A., S.F.E., R.M.K., A.M.A., R.R.S. and M.H.E.; methodology, H.M.A., A.M.A., R.R.S. and M.H.E.; software, H.M.A., A.M.A., R.R.S., D.S.H., I.A. and M.H.E.; validation, H.M.A., S.F.E., R.M.K., A.M.A., R.R.S., D.S.H., I.A. and M.H.E.; formal analysis, investigation, resources, H.M.A., A.M.A., R.R.S., D.S.H., I.A. and M.H.E.; data curation, writing—original draft preparation, H.M.A., A.M.A., R.R.S., D.S.H. and M.H.E.; writing—review and editing, H.M.A., S.F.E., R.M.K., A.M.A., R.R.S., D.S.H., I.A. and M.H.E.; visualization, H.M.A., S.F.E., R.M.K., A.M.A., R.R.S., D.S.H., I.A. and M.H.E.; supervision, project administration, H.M.A., S.F.E., R.M.K. and M.H.E. All authors have read and agreed to the published version of the manuscript.

## Data Availability

All processed data in this work are incorporated into the article.
